# A Drug Delivery Strategy: Binding Enkephalin to Asialoglycoprotein Receptor by Enzymatic Galactosylation

**DOI:** 10.1371/journal.pone.0095024

**Published:** 2014-04-15

**Authors:** Michelle P. Christie, Pavla Simerská, Freda E.-C. Jen, Waleed M. Hussein, Mohamad F. M. Rawi, Lauren E. Hartley-Tassell, Christopher J. Day, Michael P. Jennings, Istvan Toth

**Affiliations:** 1 School of Chemistry and Molecular Biosciences, The University of Queensland, St Lucia, Queensland, Australia; 2 Institute for Glycomics, Griffith University, Southport, Queensland, Australia; 3 School of Pharmacy, The University of Queensland, Pharmacy Australia Centre of Excellence, Woolloongabba, Queensland, Australia; Biological Research Centre of the Hungarian Academy of Sciences, Hungary

## Abstract

Glycosylation of biopharmaceuticals can mediate cell specific delivery by targeting carbohydrate receptors. Additionally, glycosylation can improve the physico-chemical (drug-like) properties of peptide based drug candidates. The main purpose of this study was to examine if glycosylation of the peptide enkephalin could facilitate its binding to the carbohydrate receptor, asialoglycoprotein. Firstly, we described the one-pot enzymatic galactosylation of lactose modified enkephalin in the presence of uridine-5′-diphosphogalactose 4-epimerase and lipopolysaccharyl α-1,4-galactosyltransferase. Stability experiments using human plasma and Caco-2 cell homogenates showed that glycosylation considerably improved the stability of enkephalin (at least 60% remained stable after a 2 hr incubation at 37°C). *In vitro* permeability experiments using Caco-2 cells revealed that the permeability of mono- and trisaccharide conjugated enkephalins was 14 and 28 times higher, respectively, than that of enkephalin alone (Papp 3.1×10^−8^ cm/s). By the methods of surface plasmon resonance and molecular modeling, we demonstrated that the enzymatic glycosylation of enkephalin enabled binding the asialoglycoprotein receptor. The addition of a trisaccharide moiety to enkephalin improved the binding of enkephalin to the asialoglycoprotein receptor two fold (K_D_ = 91 µM). The docking scores from molecular modeling showed that the binding modes and affinities of the glycosylated enkephalin derivatives to the asialoglycoprotein receptor complemented the results from the surface plasmon resonance experiments.

## Introduction

The clinical use of peptide-based therapeutics is mainly limited by their poor membrane permeability and stability in biological systems. Different strategies have been used to improve the physico-chemical characteristics of peptides to make them more amenable as therapeutics [1], [2]. These include chemical modifications such as cyclization and peptide bond reduction [3], use of unnatural amino acids [4], [5], co-administration with penetration enhancers such as bile salts and surfactants [6], and conjugation with bacterial and viral proteins [7], lipids [8], [9], and carbohydrates [10]–[12].

The coupling of carbohydrate moieties such as glucose or lactose to peptides improved their solubility, stability and permeability [13]–[17]. Additionally, glycosylation enhanced bioavailability and passage through the blood brain barrier [18]–[20]. The addition of carbohydrates can also enable cellular uptake via sugar transporters [11], [21] and may be used to target specific cells via carbohydrate receptors [17], [22]. The transport of glucose and galactose across the intestinal membranes occurs primarily via the sodium-glucose co-transporter 1 (SGLT1) or the facilitated diffusion glucose transporter protein 2 (GLUT2) [23]. Although these transporters are primarily responsible for the transport of sugars, some studies have implicated them in the movement of other carbohydrate-modified compounds such as glycopeptides [11], [19], [21].

Several studies used carbohydrate receptor binding to target drug molecules, DNA and peptides to specific cells types [17], [22], [24], [25]. In this study, we focused on the asialoglycoprotein receptor (ASGPR) located on hepatic cells [26] and in discrete areas of the brain, such as the cerebellum and brain stem [27]. ASGPR belongs to the C-type lectin family [28] and is targeted by glycoproteins and liposaccharides that contain terminal galactose or *N*-acetylgalactosamine, for example asialoorosomucoid [29] and gonococcal lipooligosaccharide [30], [31].

Leu-enkephalin (Tyr-Gly-Gly-Phe-Leu) is an opioid peptide with known pain-regulating activity and the potential to be used as a therapeutic when delivered to the central nervous system. Several previous attempts have been made to improve the delivery of enkephalin to the central nervous system, including lipidation [8], [9] and glycosylation [9], [32]. Various glycosyltransferases were found to be an efficient tool for the addition of carbohydrate moieties to biologically active peptides [33]–[35]. We previously galactosylated the enkephalin peptide chemically by solid phase peptide synthesis and enzymatically using galactosyltransferase as described by Simerska et al. [35].

**Figure 1:**
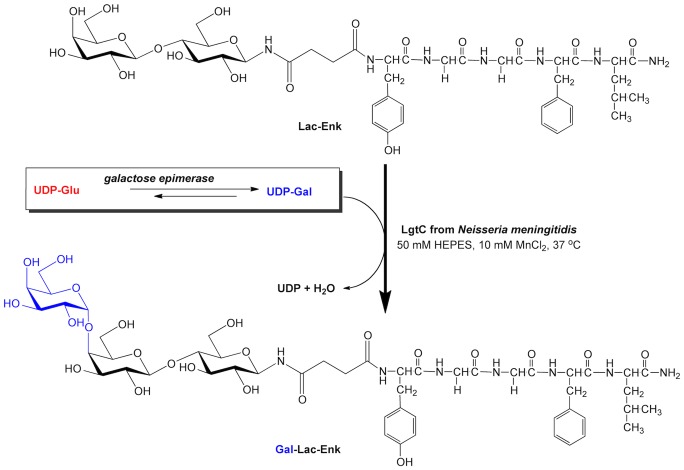
Enzymatic conversion of Lac-Enk to Gal-Lac-Enk by LgtC and galactose epimerase in the presence of UDP-Glc in 50 mM HEPES pH 7, 10 mM MnCl_2_ buffer at 37°C for 48 hrs.

The main aim of this study was to examine the ability of the glycosylated compounds *N^1^*-Leu-enkephalin-*N^4^*-(β-D-galactopyranosyl)succinamide (Gal-Enk), *N^1^*-Leu-enkephalin-*N^4^*-(β-D-galactopyranosyl-(1→4)-β-D-glucopyranosyl)succinamide (Lac-Enk) and *N^1^-*Leu-enkephalin*-N^4^*-(α-D-galactopyranosyl-(1→4)-β-D-galactopyranosyl-(1→4)-β-D-glucopyranosyl)succinamide (Gal-Lac-Enk) to bind to the carbohydrate receptor ASGPR. First, we performed a one-pot enzymatic reaction for the synthesis of Gal-Lac-Enk catalyzed by uridine-5′-diphosphogalactose 4-epimerase (EC 5.1.3.2; galactose epimerase) and lipopolysaccharyl α-1,4-galactosyltransferase (EC 2.4.1.; LgtC) using uridine-5′-diphosphate-α-D-glucose (UDP-Glc) and Lac-Enk as substrates (Figure 1). The stability and permeability of the carbohydrate-conjugated enkephalins was tested *in vitro* using Caco-2 cells. Finally, the binding of glycosylated-enkephalins to immobilized ASGPR was assessed using surface plasmon resonance (SPR) experiments and molecular docking.

## Experimental Section

### Materials

All chemicals were of analytical grade or equivalent (unless otherwise stated) and purchased from EMD (Darmstadt, Germany), Mimotopes (Clayton, Australia), Merck (Kilsyth, Australia), Sigma-Aldrich (Castle-Hill, Australia), Lab-Scan (Dublin, Ireland), Univar (Ingleburn, Australia), Scharlau (Port Adelaide, Australia), Grand Island Biological Company (GIBCO, Mulgrave, Australia), CalBioChem (Darmstadt, Germany). Rink Amide 4-methyl benzhydryl amine (Rink Amide-MBHA) was from Peptides International, USA. TALON resin used for the purification of LgtC enzyme was from Clontech (Mountain View, USA). Galactose epimerase was from Sigma Aldrich (Castle-Hill, Australia) and UDP-Glc and uridine-5′-diphosphate-α-D-galactose (UDP-Gal) was from CalBioChem (Darmstadt, Germany).

Analytical reversed phase high performance liquid chromatography (RP-HPLC) was conducted on Shimadzu Instrumentation (Kyoto, Japan) with LabSolutions software, SIL-20AC HT autosampler, LC-20AB pump, SPD-M10A detector and a DGU-20A5 degasser. Either an Alltima C18 column (5 µm 4.6 mm×250 mm; Grace Davison Discovery Sciences, Deerfield, USA) or a Grace Vydac C18 column (10 µm, 4.6 mm×250 mm; Grace Davison Discovery Sciences, Deerfield, USA) was used. Solvent A was 0.1% trifluoroacetic acid (TFA) in water and solvent B was 90% acetonitrile (ACN)/water/0.1% TFA. Samples were run for 30 min at a flow rate of 1 mL/min with detection at 214 nm.

Preparative RP-HPLC was conducted on a Waters Delta 600 system (Milford, USA) at a flow rate of 20 mL/min and detection at 230 nm using PicoLog software. A Vydac C18 column (10 µm, 22 mm×250 mm; Grace Davison Discovery Sciences, Deerfield, USA) was used for all purifications.

Electrospray ionization mass spectrometry (ES-MS) and liquid chromatography mass spectrometry (LC-MS) were conducted on a Perkin-Elmer-Sciex API 3000 mass spectrometer (Applied Biosystems/MDS Sciex, Toronto, Canada). Solvents used for ES-MS and LC-MS consisted of solvent A: 0.1% acetic acid (AcOH) in water and solvent B: 90% ACN/10% water/0.1% AcOH. A Phenomenex Luna C18 column (5 µm, 50×2.0 mm, Torrance, USA) was used for LC-MS analysis. Instrument control, data acquisition, and data analysis were carried out using Analyst software version 1.4.1. (Applied Biosystems/MDS Sciex, Toronto, Canada).

Human colorectal adenocarcinoma cells (Caco-2 cells) were obtained from the American Type Culture Collection, Rockville, USA. A Branson Sonifier 250 (Branson Ultrasonics, Danbury, USA) was used to lyse the cells for stability experiments. Protein content was measured using the BioRad protein assay kit (BioRad, Hercules, USA). TPP 96-well plates for stability assays were purchased from Sigma Aldrich (Castle Hill, Australia). For permeability studies Caco-2 cell monolayers were grown in 24 well plates with Transwell polycarbonate cell culture inserts (pore size 0.4 µm, diameter 6.5 mm) from Corning Incorporated (New York, USA). A Millicell-ERS epithelial Transep volt-ohmmeter system (EMD Millipore Corporation, Billerica, USA) was used to measure the transepithelial electrical resistance (TEER) values of the cells. Scintillation counting was carried out using Liquid Scintillation Systems from Beckman Coulter (Brea, USA).

SPR experiments were performed on a Biacore T100 (GE Healthcare, Little Chalfont, UK) at 25°C. Recombinant human ASGPR (the subunit of ASGPR that contains the carbohydrate binding site) used was from Sino Biological Inc. (Beijing, China). The receptor was immobilized onto a series S sensor chip CM5 (GE Healthcare, Little Chalfont, UK) using the *N*-hydroxysuccinimide (NHS) capture kit (GE Healthcare, Little Chalfont, UK).

### Chemo-enzymatic Synthesis of Enkephalin and its Glycopeptides

Enkephalin and carbohydrate-modified enkephalins, Gal-Enk, Lac-Enk and Gal-Lac-Enk, were prepared according to Simerska et al. [35]. Peptides and glycopeptides were assembled using standard solid phase peptide synthesis on Rink Amide-MBHA resin using Fmoc chemistry protocols. Crude peptide and glycopeptides were purified by preparative RP-HPLC, a gradient of 0–100% solvent B over 60 min was used for enkephalin and a gradient of 20–35% solvent B over 60 min was used for the glycosylated enkephalins. Fractions that contained pure peptide/glycopeptide were analyzed using analytical RP-HPLC and ES-MS and corresponded with those previously published [35].

The galactosyltransferase LgtC was recombinantly expressed in *Escherichia coli* AD202 and purified using TALON metal affinity resin [35]. The purified protein was dialyzed into 20 mM Na_2_HPO_4_ buffer at pH 7.5, concentrated to 2 mg/mL and stored in 50% glycerol. One-pot enzyme reaction was performed using 6 mM UDP-Glc (20 µL), and 2 mM Lac-Enk (20 µL) in 20 mM 4-(2-hydroxyethyl)-1-piperazineethanesulfonic acid (HEPES) buffer containing 10 mM MnCl_2_ at 37°C with 0.2 U (0.015 mg) of galactose epimerase and 1 µL of LgtC (2 mg/mL). The positive control reaction contained 6 mM UDP-Gal (20 µL), and 2 mM Lac-Enk (20 µL) in 20 mM HEPES buffer and 1 µL of LgtC (2 mg/mL), while the negative control contained 6 mM UDP-Glc (20 µL), and 2 mM Lac-Enk (20 µL) in 20 mM HEPES buffer and 1 µL of LgtC (2 mg/mL). An additional 10 µL of UDP-Glc, 0.2 U of galactose epimerase, and 1 µL of LgtC were added to the respective reaction mixtures after 24 hrs. The enzymatic reactions were incubated for a further 24 hrs at 37°C. Reaction progress was followed by RP-HPLC using an Alltima C18 with a gradient of 0–90% solvent B. Gal-Lac-Enk product formation was confirmed by ES-MS (C_52_H_76_N_8_O_23_, 1139.5) m/z 1140.5 [M+H]^+^ (calcd. 1140.5) and RP-HPLC (Alltima C18, 0–90% B) t_R_ = 16.8 min.

### Plasma Stability Assay

Blood sample collection from a healthy and consenting human adult for this study was approved by and conducted according to the guidelines set by The University of Queensland Medical Research Ethics Committee (approval number: 2009000661). Written consent was obtained prior to the collection of blood samples. Plasma was isolated from fresh-heparin treated blood by centrifugation at 660×*g* for 15 min. A 1 mg/mL solution of each test compound (enkephalin, Gal-Enk, Lac-Enk, and Gal-Lac-Enk) was prepared in phosphate buffered saline (PBS). Compounds (300 µL) were mixed with 300 µL of the plasma, pre-warmed at 37°C and incubated at 37°C. Samples (50 µL) were collected at predetermined time points (0, 5, 10, 20, 30, 40, 60, 90 and 120 min), and added into 75 µL ACN to precipitate the plasma proteins. Samples were then centrifuged at 7300×*g* for 10 min. The resultant supernatant (30 µL) was analyzed by analytical RP-HPLC for the amount of test compound remaining in the solution using an elution gradient of 20–35% (enkephalin) or 0–70% solvent B (glycosylated enkephalins) over 30 min on a C18 Vydac column.

### Caco-2 Cell Stability Assay

Caco-2 cells cultured for 21 to 28 days were washed 3 times with 5 mL of 0.02% ethylenediaminetetraacetic acid solution. They were detached using a cell scraper and re-suspended in 4 mL of Hank’s buffered salt solution (HBSS) buffered with 25 mM HEPES to pH 7.4 (HBSS–HEPES). Cells were lysed by sonication on a Branson Sonifier, four times for 5 sec, with an output of 2 and 20% duty cycle. The cell lysate was then centrifuged at 430×*g* for 5 minutes to remove cell debris. The protein content of the supernatant was determined using the BioRad protein assay (Gibbons et al. 1990) and adjusted to 0.5–0.8 mg/mL with HBSS-HEPES. The test compounds were solubilized in HBSS-HEPES buffer and 100 µL samples (100 µM) were mixed with 100 µL of the Caco-2 cell homogenate in a TPP 96-well plate and incubated at 37°C with shaking. Each compound was tested in triplicate at 5, 10, 20, 30, 40, 50, 60 and 90 minutes. At each time point, 10 µL of sample was mixed with ACN/water solution containing 5.5% TFA to stop enzymatic degradation. Collected samples were analyzed using LC-MS in positive ion electrospray mode and selective ion monitoring. A Phenomenex Luna C18 column with a linear gradient of 0–80% solvent B was used for enkephalin and a linear gradient of 0–70% solvent B for all other compounds over 5.5 min at a 0.5 mL/min flow rate. A standard curve was generated for each compound and used to calculate the compound’s concentration.

### Caco-2 Cell Permeability Assay

A cell suspension of Caco-2 cells in the cell culture medium was adjusted to a cell density of 1×10^6^ cells/mL before being aliquoted (100 µL) into Transwell polycarbonate cell culture inserts in a 24-well plate, while 600 µL of media was added to the basolateral chamber. The culture medium was changed every other day for 21–28 days. TEER values were measured before and after the assay, to determine the integrity of tight junctions of the cell monolayers. TEER values of the monolayers were between 730 - 975 Ωcm^2^. The integrity of the monolayers was further monitored by measuring the permeability of radiolabelled [^14^C]-D-mannitol (0.09 mCi/mL in 90% ethanol in water) by adding a 100 µL solution of 1.80 µCi, 32.73 nmol/4 mL solution in HBSS–HEPES buffer to the apical chamber of three wells. Cell monolayers were washed with pre-warmed HBSS–HEPES and incubated for 30 min at 37°C. Test compounds and the control propanolol, were prepared in HBSS–HEPES buffer to a final concentration of 200 µM. After incubation, buffer in the apical chamber was removed and replaced with 100 µL aliquots of each of the test compounds. At selected time points (30, 90, 120, and 150 min) 0.4 mL of buffer was collected from the basolateral chambers and replaced with the same volume of buffer. The concentration of the peptide in the collected samples was determined using LC-MS analysis as described for the stability assay above. Radioactivity of the [^14^C]-D-mannitol samples was quantified by liquid scintillation counting (Liquid Scintillation Systems).

The apparent permeability (P*_app_*, cm/s) of each compound was calculated using the following equation:
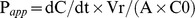



Where dC/dt is the steady-state rate of change in the test item concentration (M/s) or radiochemical concentration (dpm mL/s) in the receiver chamber, Vr is the volume of the receiver chamber (mL), A is the surface area of the cell monolayers and C0 is the initial concentration in the donor chamber (M or dpm/mL).

Permeability experiments (as described above) were conducted to investigate whether the compounds used the sugar transporters SGLT1 or GLUT2 for their transport across cell membranes. Samples containing 200 µM test compound and 100 µM phlorizin (inhibitor for SGLT1) or 100 µM phloretin (inhibitor for GLUT2) in HBSS-HEPES buffer were added to the apical chamber. Solution (0.4 mL) was collected from the basolateral chambers and replaced with the same volume of buffer after 30, 90, 120, and 150 min incubations. *P*_app_ values for each compound were then calculated as above.

The average P*_app_* values (±S.D.) from three replicates is presented. Unpaired t-tests (p<0.05) were performed using Prism software (Version 6, Graphpad Software Inc., La Jolla, USA) to determine whether the P*_app_* values between different compounds were statistically significant.

### SPR Analysis of ASGPR Binding

The affinities of enkephalin derivatives for ASGPR were tested using SPR methodology on a Biacore T100 at 25°C. Recombinant human ASGPR was immobilized onto a series S sensor chip CM5 using the NHS capture kit, where ASGPR was covalently linked to the surface of the chip via free amine groups. Compounds were tested at 10–1000 µM concentrations using multi-cycle kinetics with at least three experiments performed for each interaction. Single cycle kinetics was applied to optimize concentrations prior to completion of multi-cycle kinetics as described by Dumont et al. [36]. D-Galactose and D-lactose at the same concentrations as the test compound were used as positive controls. The running buffer for all SPR experiments was 20 mM HEPES, pH 7.4, 150 mM NaCl, 5 mM CaCl_2_, and 0.005% TWEEN 20.

### Molecular Modeling Studies Using MolDock

*In silico* docking was used to investigate the binding affinity of D-galactose, D-lactose and enkephalin derivatives to ASGPR based on energy minimization calculated by Molegro Virtual Docker software [37]. The crystal structure of ASGPR (PDB code: 1DV8, [38]) was used as the target in this study. Binding affinities of enkephalin, Gal-Enk, Lac-Enk, and Gal-Lac-Enk for ASGPR were displayed by docking scores [39].

## Results and Discussion

### Chemo-enzymatic Synthesis of Enkephalin and its Glycosylated Derivatives

**Figure 2:**
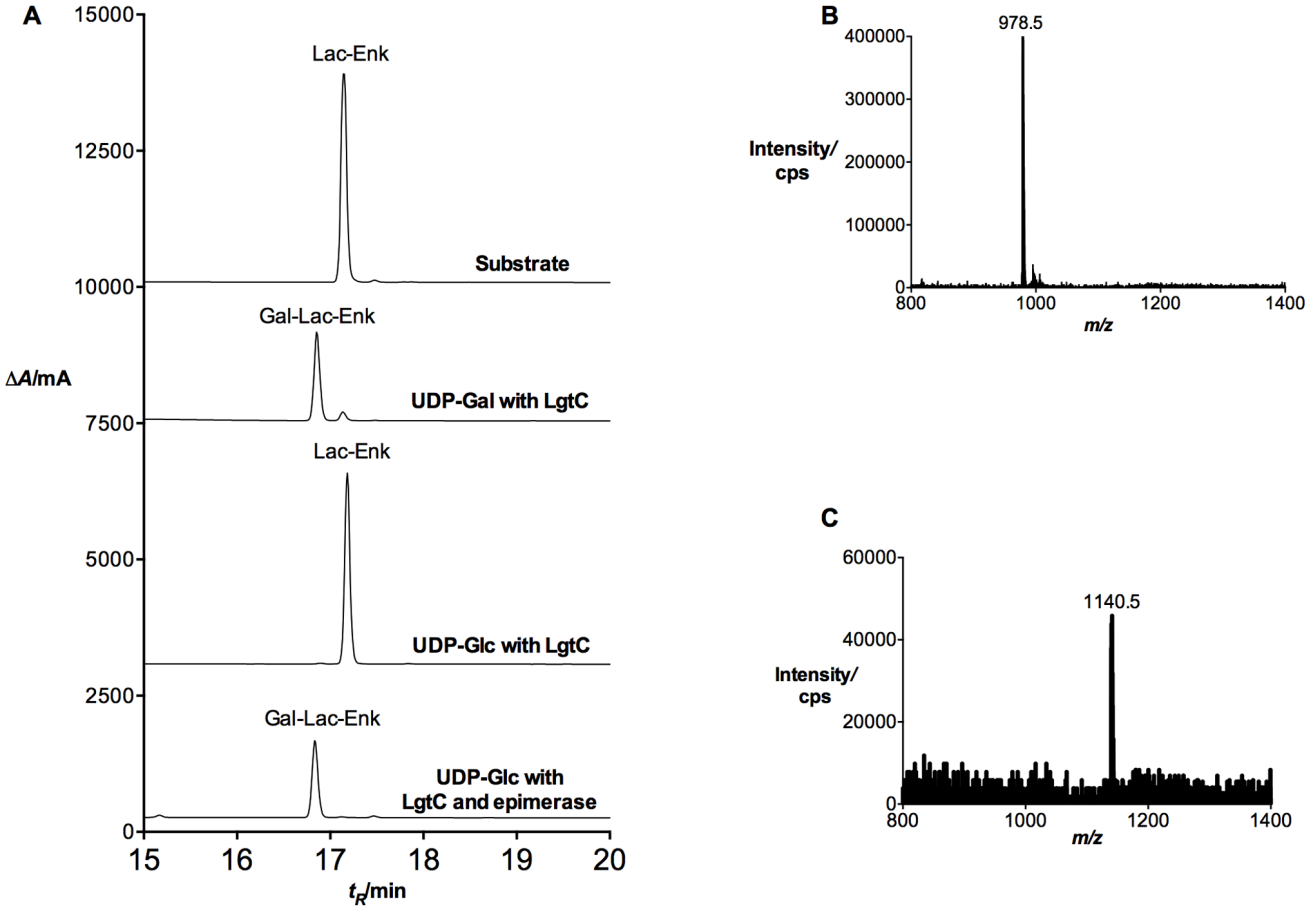
Enzymatic transformation of Lac-Enk to Gal-Lac-Enk. **A)** RP-HPLC analysis of one-pot reaction for Lac-Enk with UDP-Glc and galactose epimerase. From top to bottom: substrate (Lac-Enk) only; positive control – reaction mixture containing UDP-Gal and LgtC; negative control – reaction mixture containing UDP-Glc and LgtC; reaction mixture containing UDP-Glc, LgtC and galactose epimerase. All reactions were conducted in 50 mM HEPES (pH 7), 10 mM MnCl_2_ buffer at 37°C. RP-HPLC was conducted on an Alltima C18 column using a gradient of 0–90% solvent B, t_R_ = 17.2 min (substrate, Lac-Enk) and t_R_ = 16.8 min (product, Gal-Lac-Enk). **B)** ES-MS analysis of the acceptor, Lac-Enk m/z (C_44_H_63_N_7_O_18_, 977.4) m/z 978.5 [M+H]^+^ (calcd. 978.4). **C)** ES-MS analysis of the product, Gal-Lac-Enk (C_52_H_76_N_8_O_23_, 1139.5) m/z 1140.5 [M+H]^+^ (calcd. 1140.5).

The chemo-enzymatic synthesis of Gal-Lac-Enk was previously conducted using the lipopolysaccharyl galactosyltransferase LgtC with UDP-Gal as a donor [35]. However, the high cost of UDP-Gal limited the large-scale use of this reaction (despite its high efficiency). On the other hand, UDP-Glc is available for a lower cost and is readily converted to UDP-Gal by galactose epimerase (Figure 1). Previous studies successfully used galactose epimerase for the synthesis of UDP-Gal from UDP-Glc for other one-pot reactions [40]–[42]. A similar one-pot synthesis of Gal-Lac-Enk was attempted with UDP-Glc and galactose epimerase to synthesize UDP-Gal, which in turn acted as a donor for the LgtC-catalyzed transfer of a galactose unit to Lac-Enk to produce Gal-Lac-Enk (Figure 1). Reaction progress was monitored by RP-HPLC and ES-MS (Figure 2), which showed product formation in the reaction catalyzed by galactose epimerase and LgtC after 48 hrs of incubation at 37°C. The control reaction using UDP-Glc and LgtC only (without galactose epimerase) did not show any product formation.

### Stability of Carbohydrate-modified Enkephalins

**Figure 3:**
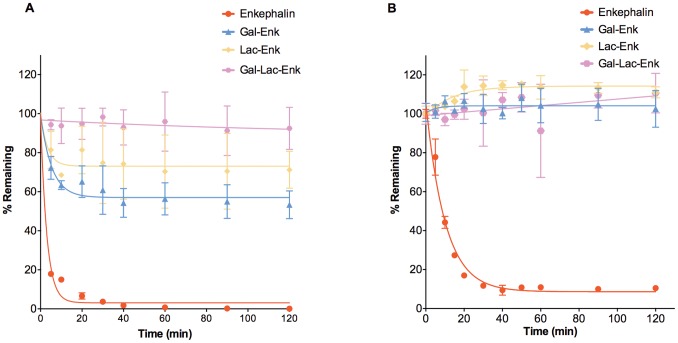
Stability profiles of enkephalin and carbohydrate-derived enkephalins. **A)** Plasma stability assay: the concentration of compounds in plasma was determined at various time points using RP-HPLC on a C18 Vydac column, gradient of 20–35% (enkephalin) or 0–70% solvent B (glycosylated enkephalins) over 30 min. **B)** Caco-2 cell stability assay: The concentration of compound in Caco-2 cell homogenates at various time points was quantified using LC-MS. Data presented is the mean ± S.D. (n = 3).

Enkephalin is susceptible to degradation by enzymes such as aminopeptidases, carboxypeptidases and endopeptidases, leading to rapid degradation *in vitro* [43], [44]. In this study, we used human plasma and Caco-2 cells to test the stability of the glycosylated enkephalins. Plasma stability experiments were conducted to determine the stability of the compounds upon entry into the circulatory system. Compounds with low plasma stability generally showed rapid clearance and consequently low efficacy *in vivo* [45]. Caco-2 cells are derived from human carcinoma cells and have been used to assess enzymatic stability upon oral or systemic delivery [46]. Our results from plasma and Caco-2 stability experiments showed that enkephalin had a half-life of 2.1 min and 7.1 min, respectively. These values are similar to previously reported values for enkephalin [47], [48]. In contrast, the glycosylated enkephalins were considerably more stable. Around 60, 70 and 100% of Gal-Enk, Lac-Enk and Gal-Lac-Enk, respectively, remained in human plasma even after incubation at 37°C for 120 min (Figure 3A). The stability profiles of the glycopeptides (Gal-Enk and Lac-Enk) plateaued after a 40 min incubation in human plasma. This is most likely to have occurred because the test compounds saturated the peptidases found in plasma. The saturation effect was observed for the glycosylated enkephalins even when a lower concentration of test compound (0.3 mg/mL) was used (data not shown). We were therefore unable to obtain an accurate measure of the half-life of glycosylated enkephalins in plasma. In Caco-2 cell homogenates, the glycosylated compounds remained stable throughout the course of the experiment (Figure 3B). The increased stability observed for the glycosylated peptides over the parent peptide can be attributed to increased protection of the peptide from enzymes such as aminopeptidase. It is likely that the presence of the carbohydrate moiety sterically hindered the peptidases from accessing the enkephalin peptide. Peptides such as endomorphin [11], luteinizing hormone-releasing hormone [16] and enkephalin modified at the N-terminus [48], also showed a greatly increased stability in comparison to the parent peptides *in vitro*. For example, Wang et al. [48] observed that the modification of the N-terminus of enkephalin with a lipidic moiety increased the half-life of the parent peptide from 6.7 min to ∼193 hrs.

### Permeability of Carbohydrate-modified Enkephalins

The permeability of enkephalin and glycosylated enkephalins was determined *in vitro* using Caco-2 cells. The apparent permeability of the negative control ^14^C mannitol was 4.4×10^−9^ (±3.86) cm/s and the apparent permeability for the positive control propanolol was 1.34×10^−4^ (±0.009) cm/s.

**Figure 4:**
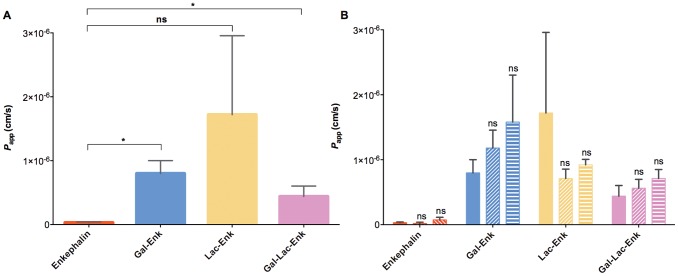
Permeability studies of enkephalin and carbohydrate-derived enkephalins. **A)** Apparent permeability (*P*_app_ (cm/s) of enkephalin and glycosylated enkephalins. (p value <0.05) **B)** Apparent permeability of enkephalin and glycosylated enkephalin in the presence of the SGLT1 inhibitor phlorizin (horizontal lines) and the GLUT2 inhibitor phloretin (diagonal lines). *P*_app_ values in the presence of inhibitor was compared to that of compound in the absence of any inhibitor (p value <0.05) Data reported is the mean ± S.D. (n = 3).

Enkephalin displayed an apparent permeability of 3.1×10^−8^ (±1.1) cm/s. The presence of a mono- or a tri-saccharide moiety in the glycosylated enkephalin structure increased the apparent permeability of enkephalin by 28 and 14 fold, respectively (p<0.05, Figure 4A, Table 1). The permeability of Lac-Enk (*P*_app_ 1.72×10^−6^ (±1.24) cm/s) was also higher than that of enkephalin alone. However, this increase was not statistically significant due to the high standard deviation observed for the Lac-Enk sample (Figure 4A). Although all the glycosylated enkephalins were more permeable than the parent peptide, we did not observe any significant difference between them. Wong et al. [12] reported that enkephalin was degraded on the apical side of the cells in Caco-2 permeability experiments and consequently reduced the apparent permeability of the compound. The increased permeability of glycosylated enkephalins may result from their increased metabolic stability (Table 1).

**Table 1:** The apparent permeability (*P*_app_) of enkephalin and glycosylated enkephalins, assessed using *in vitro* Caco-2 cell monolayer experiments (n = 3).

	*P*_app_ (×10^−7^ cm/s)		
Compound	Control (compound only)	Phlorizin	Phloretin
Enkephalin	0.31±0.11	0.19±0.19	0.74±0.40
Gal-Enk	7.98±2.03	11.80±2.76	15.80±7.23
Lac-Enk	17.20±12.40	7.11±1.45	9.21±0.84
Gal-Lac-Enk	4.40±1.63	5.61±1.37	7.12±1.37

The higher permeability observed for the glycosylated enkephalins could also be due to improved transport across the Caco-2 cell membrane. The compounds may be transported into cells via passive diffusion, transporter molecules or by transcytosis. Some reports showed that small glycosylated peptides moved across the cell membrane via sugar transporters [21]. Transport by absorptive transcytosis was also suggested for glycosylated opioid peptides such as enkephalin [18], [49]. The apparent permeability of these compounds was measured in the presence of SGLT1 and GLUT2 sugar transport inhibitors; phlorizin and phloretin, respectively [23], [50]–[52], to test if the carbohydrate-derived enkephalins were able to utilize sugar transporters to enter cells. Our experiments demonstrated that none of the glycosylated enkephalins were inhibited either by phlorizin or phloretin (Table 1, and Figure 4B) suggesting that transport across membranes was not mediated by SGLT1 or GLUT2. Adsorptive transport is therefore the most likely mechanism by which the carbohydrate-derived enkephalin compounds crossed the membrane.

### SPR Experiments to Determine Binding between ASGPR and Enkephalin Derivatives

Peptide glycosylation could facilitate their binding to specific carbohydrate receptors [17], [22]. All of our glycosylated products contained a terminal galactose moiety making them potential targets for the lectin receptor, ASGPR. SPR experiments with ASGPR protein immobilized onto the sensor chip were performed to test whether the glycosylated enkephalins could bind to ASGPR. D-Galactose and D-lactose were used as controls to show that ASGPR could actively bind its native substrate while bound to the sensor chip. Enkephalin, Gal-Enk, and Lac-Enk showed similar binding to ASGPR with K_D_ values of ∼200 µM (Table 2). Our results indicated that the increasing length of the glycan residue resulted in higher binding affinity to the receptor. Consequently, Gal-Lac-Enk, which contained a trisaccharide moiety, displayed a two-fold increase in binding affinity (K_D_ 91.1 µM) compared to the binding affinity of the peptide alone. The interaction kinetics was measured to confirm that binding to ASGPR was biologically relevant. Kinetic analysis demonstrated that enkephalin modified with longer glycan chains (Lac-Enk and Gal-Lac-Enk) had a slower on-rate (K_on_) and a slower off-rate (K_off_) (Table 2). However, the off-rate of Lac-Enk for dissociating from ASGPR was twice that of Gal-Lac-Enk resulting in the lower affinity (K_D_ 211.3 µM) reported for Lac-Enk. This indicated that it took longer for Gal-Lac-Enk to enter the binding pocket of ASGPR but once Gal-Lac-Enk was bound it was more stable than Lac-Enk, Gal-Enk, or enkephalin. Together the data suggested that only Gal-Lac-Enk binding to ASGPR was biologically relevant and therefore glycosylation of enkephalin with at least a trisaccharide moiety was necessary to achieve effective ASGPR binding.

**Table 2:** Kinetics and affinity analysis of enkephalin and glycosylated enkephalins with ASGPR.#

	K_on_ (1/M.s)	K_off_ (1/s)	K_D_ (µM)
Enkephalin	349.7±69.8	0.0970±0.0290	277.4±96.0
Gal-Enk	328.3±84.8	0.0720±0.0250	219.3±164.0
Lac-Enk	40.7±6.3	0.0086±0.002	211.3±70.9
Gal-Lac-Enk	45.0±28.9	0.0041±0.003	91.1±22.1
D-Galactose	ND	ND	59.7±30.1
D-Lactose	ND	ND	19.2±3.28

# ND: Kinetics values for D-galactose and D-lactose could not be uniquely determined due to size differences between the analyte (ASGPR, 37 kDa) and the ligand (D-galactose and D-lactose 180 and 342 Da, respectively) resulting in small RU values and larger than acceptable Chi^2^ values for the kinetic analysis. Calculation of affinity was not affected.

### Molecular Modeling Studies for Enkephalin Derivatives Binding to ASGPR

We also conducted molecular modeling experiments for the enkephalin derivatives using the crystal structure of ASGPR (PDB 1DV8, [38]). The molecular docking scores obtained showed a good correlation to the surface plasmon resonance data (Table 2). Enkephalin, Gal-Enk and Lac-Enk showed very low binding affinities to the active site of ASGPR with positive MolDock scores (Table 3). However, Gal-Lac-Enk bound to ASGPR with a lower total interaction energy (a negative docking score of −9 kcal/mol) indicating a higher binding affinity to the receptor (Table 3).

**Table 3:** Calculated binding affinities using MolDock for enkephalin and its derivatives with ASGPR.

Compound	MolDock Score# (kcal/mol)
Enkephalin	Positive
Gal-Enk	Positive
Lac-Enk	Positive
Gal-Lac-Enk	−9
D-Galactose	−34
D-Lactose	−45

# The binding affinity was calculated by energy minimization and given a MolDock score, which was derived from the piecewise linear potential scoring function [53].

**Figure 5:**
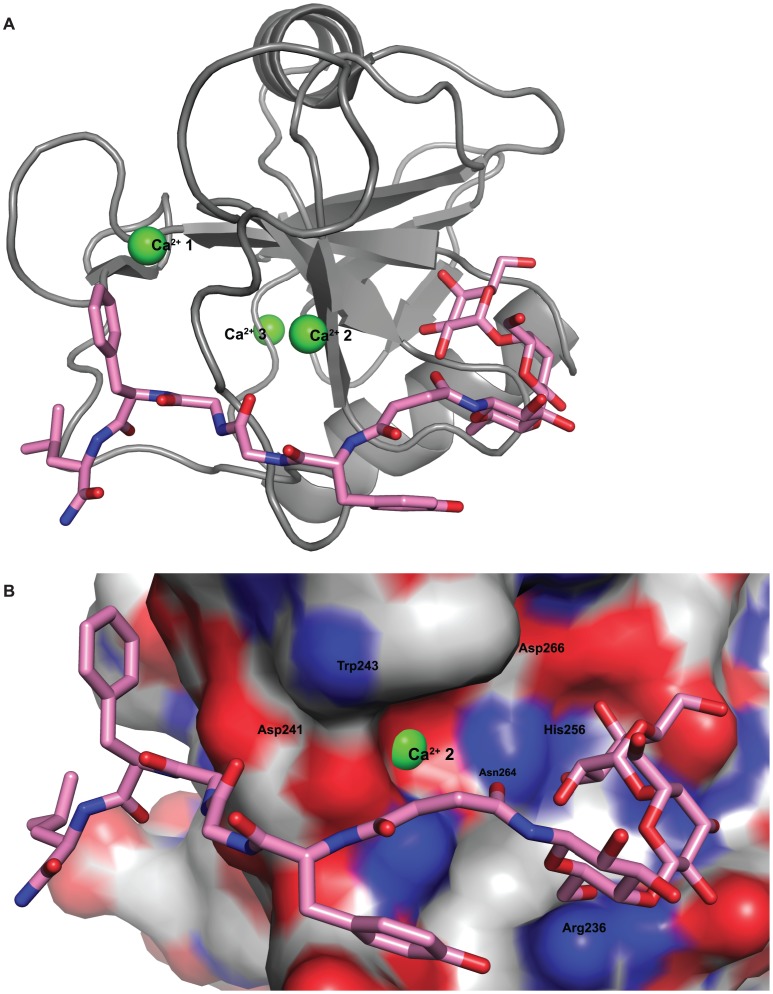
Docking between Gal-Lac-Enk and the ASGPR receptor. **A)** Cartoon representation of Gal-Lac-Enk with the highest negative docking score conformation, in the active site of ASGPR. **B)** Surface view of ASGPR with Gal-Lac-Enk, docked into the active site. For clarity, atoms have been colored as follows: blue-nitrogen, red-oxygen, gray-carbon, green-Ca^2+^ (on ASGPR) and pink-carbon (on Gal-Lac-Enk).

**Figure 6:**
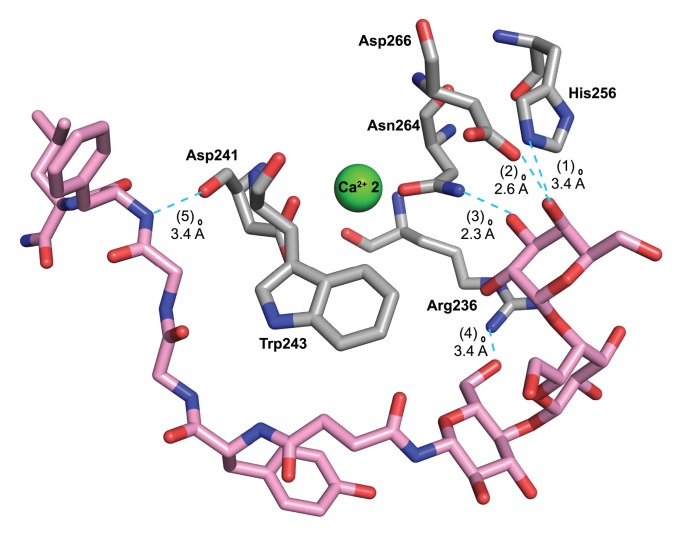
Details of the interaction between Gal-Lac-Enk in the conformation that gave the highest negative docking score and the ASGPR receptor. (1) A hydrogen bond between the oxygen of the -OH group on C4 of the terminal galactose moiety in Gal-Lac-Enk and the -NH group of His256 of ASGPR (O-N distance 3.4 Å), (2) a hydrogen bond between the oxygen of the -OH group on C4 of the terminal galactose of Gal-Lac-Enk and the -OH of a carboxyl group of Asp266 of ASGPR (O-O distance 2.6 Å), (3) a hydrogen bond between the oxygen of the -OH group on C3 of the terminal galactose of Gal-Lac-Enk and the NH of Asn264 of the receptor (O-N distance 2.3 Å), (4) a hydrogen bond between the oxygen of the -OH group on C6 of the glucose moiety of Gal-Lac-Enk and the -NH of Arg236 of ASGPR (O-N distance 3.4 Å) and (5) a hydrogen bond between the -NH group of Phe in Gal-Lac-Enk and the oxygen of the carbonyl group of Asp241 of ASGPR (N-O distance 2.7 Å). Atom colors are as follows: blue-nitrogen, red-oxygen, gray-carbon, green-Ca^2+^ (on ASGPR) and pink-carbon (on Gal-Lac-Enk). Hydrogen bonds are represented in cyan.

The similar outcomes from both the SPR experiment and the molecular modeling studies indicated that the attachment of enkephalin most likely hindered the terminal galactose moiety on Gal-Enk and Lac-Enk from accessing the active site of ASGPR. In the case of Gal-Lac-Enk that contained a trisaccharide moiety, the greater distance between peptide and the terminal galactose enabled binding to the active site of the receptor (Figure 5 and 6).

## Conclusions

We successfully used a one-pot enzymatic reaction that contained UDP-Glc, galactose epimerase and LgtC galactosyltransferase to galactosylate Lac-Enk to Gal-Lac-Enk. In comparison to the parent peptide, all chemo-enzymatically glycosylated enkephalins showed improved stability and permeability *in vitro*. The interaction between the glycosylated enkephalins and the carbohydrate receptor ASGPR was examined by SPR experiments and molecular modeling analysis. The SPR systems are widely used as a standard tool in areas such as pharmaceutical drug discovery. Here, SPR in conjunction with molecular modeling, allowed the rapid *in vitro* evaluation of glycosylated peptide-receptor interactions. The SPR results showed that the addition of a glycan moiety to the enkephalin peptide enabled binding of the peptide to ASGPR. The binding affinity between ASGPR and glycosylated enkephalins increased when the number of sugar moieties increased from one (Gal) to three (Gal-Lac). The molecular docking analysis also showed the binding of a trisaccharide (Gal-Lac) modified enkephalin to the active site of ASGPR. The chemo-enzymatic conjugation of oligosaccharides to therapeutic peptides can potentially be applied to cell-specific targeting via carbohydrate receptors. However, further experiments, including *in vivo* experiments will be required to confirm this observation. Given the wide array of glycosyltransferases available, we envisage that this technique may also be more widely applied to modify therapeutic peptides to contain different oligosaccharide moieties for targeting other carbohydrate receptors.
